# Ergonomic risks and musculoskeletal pain in hospital cleaning workers: Convergent Care Research with mixed methods ^*^

**DOI:** 10.1590/1518-8345.7048.4176

**Published:** 2024-06-17

**Authors:** Emanuelli Mancio Ferreira da Luz, Oclaris Lopes Munhoz, Patrícia Bitencourt Toscani Greco, José Luís Guedes dos Santos, Silviamar Camponogara, Tânia Solange Bosi de Souza Magnago

**Affiliations:** 1 Universidade Federal do Rio Grande, Escola de Enfermagem, Rio Grande, RS, Brazil.; 2 Universidade Federal de Santa Catarina, Departamento de Enfermagem, Florianópolis, SC, Brazil.; 3 Universidade Federal de Santa Maria, Departamento de Enfermagem, Santa Maria, RS, Brazil.; 4 Scholarship holder at the Conselho Nacional de Desenvolvimento Científico e Tecnológico (CNPq), Brazil.

**Keywords:** Nursing, Occupational Health, Musculoskeletal Pain, Ergonomics, Housekeeping Hospital, Occupational Risks, Enfermería, Salud Laboral, Dolor Musculoesquelético, Ergonomía, Servicio de Limpieza en Hospital, Riesgos Profesionales, Enfermagem, Saúde Ocupacional, Dor Musculoesquelética, Ergonomia, Zeladoria Hospitalar, Riscos Ocupacionais

## Abstract

**Method:**

Convergent Care Research, with data production designed using mixed methods, implemented with 149 hospital cleaning workers. The methodological strategy of the convergent parallel project was employed, using observation, photographic records, questionnaires and convergence groups. The results were integrated through joint display. Data analysis with descriptive and inferential statistics and content analysis.

**Results:**

the mixing of data highlighted the multifactorial nature of exposure to ergonomic risks (uncomfortable work postures; repetitive movements; prolonged orthostatism; use of equipment not adapted to the psychophysiological needs of workers) and musculoskeletal pain in the population investigated. The latter was prevalent in the lumbar spine, ankles or feet, wrists or hands, thoracic spine and shoulders. The concept of ergonomic risk was expanded and was influenced by the psychosocial aspects of work.

**Conclusion:**

the workers investigated are exposed to modifiable multifactorial ergonomic risks related to musculoskeletal pain. It is possible to promote innovations and teaching-learning actions to minimize them, such as the continuing education program, collectively constructed with recommendations for improvements.

## Abstract (es)

**Método:**

investigación Convergente Asistencial, con producción de datos diseñada mediante métodos mixtos, realizada con 149 trabajadores de limpieza hospitalaria. Se utilizó la estrategia metodológica del proyecto paralelo convergente, basada en observación, registros fotográficos, cuestionarios y grupos de convergencia. Los resultados se integraron mediante *joint display* . Análisis de datos con estadística descriptiva e inferencial y análisis de contenido.

**Resultados:**

la combinación de datos puso de relieve la naturaleza multifactorial de la exposición a riesgos ergonómicos (posturas de trabajo incómodas; movimientos repetitivos; ortostatismo prolongado; uso de equipos no adaptados a las necesidades psicofisiológicas de los trabajadores) y al dolor musculoesquelético en la población investigada. Este último prevalecía en la columna lumbar, tobillos o pies, puños o manos, columna torácica y hombros. El concepto de riesgo ergonómico se amplió y estuvo influenciado por los aspectos psicosociales del trabajo.

**Conclusión:**

los trabajadores investigados están expuestos a riesgos ergonómicos multifactoriales modificables relacionados con el dolor musculoesquelético. Es posible promover innovaciones y acciones de enseñanza-aprendizaje para minimizarlos, como el programa de educación continua, construido colectivamente con recomendaciones de mejora.

## Abstract (pt)

**Método:**

pesquisa Convergente Assistencial, sendo a produção de dados delineada com métodos mistos, realizada com 149 trabalhadores de limpeza hospitalar. Utilizou-se a estratégia metodológica do projeto paralelo convergente, baseada em observação, registros fotográficos, questionários e grupos de convergência. Os resultados foram integrados por meio de *joint display* . Análise de dados com estatística descritiva e inferencial e análise de conteúdo.

**Resultados:**

a mixagem dos dados evidenciou o caráter multifatorial da exposição aos riscos ergonômicos (posturas de trabalho desconfortáveis; movimentos repetitivos; ortostatismo prolongado; utilização de equipamentos não adaptados às necessidades psicofisiológicas dos trabalhadores) e à dor musculoesquelética na população investigada. Esta última foi prevalente na coluna lombar, tornozelos ou pés, punhos ou mãos, coluna torácica e ombros. O conceito de risco ergonômico foi ampliado e sofreu influência dos aspectos psicossociais do trabalho.

**Conclusão:**

os trabalhadores investigados estão expostos aos riscos ergonômicos multifatoriais modificáveis relacionados à dor musculoesquelética. É possível promover inovações e ações de ensino-aprendizagem para minimizá-los, como o programa de educação continuada, construído coletivamente com recomendações de melhorias.

## Introduction

 Ergonomic risk is defined as any factor that interferes with the psychophysiological characteristics of workers, causing discomfort or health problems. Among the factors are: heavy lifting, excessive work pace, repetitive movements and incorrect working posture ^(^ 1 ^)^ . 

 When applied correctly, ergonomics enables changes in working conditions and the working environment through adaptations to jobs and work processes, with a view to improving workers’ quality of life ^(^ 1 ^-^ 2 ^)^ . This field of knowledge is not limited to the analysis of activities with machines or equipment, it also includes the risks that exist in the physical environment, in addition to cognitive, behavioral and organizational aspects ^(^ 2 ^)^ . Nevertheless, the lack of ergonomics predisposes the occurrence of occupational pathologies, such as musculoskeletal disorders, known as cumulative traumatic disorders, with consequent harm to the worker and the institution, resulting from absenteeism and sick leave ^(^ 3 ^)^ . 

 In this context, there is musculoskeletal pain (MSP), resulting from excessive use of the musculoskeletal system, associated with limited recovery time. It is expressed by reports from individuals who experience symptoms such as pain (in the musculoskeletal system), fatigue, numbness, paresthesia and limitation of movement ^(^ 3 ^)^ . This is an important public health problem and one of the main conditions, acute or chronic, in occupational groups exposed to strenuous physical demands and repetitive movements ^(^ 4 ^)^ . 

 Among these groups are workers from the Hospital Cleaning Service (SHL, for its acronym in Portuguese), who compose the support service, defined as non-health care services responsible for technical and logistical support. These workers are often subjected to precarious employment relationships, with high rates of musculoskeletal illness ^(^ 5 ^)^ . 

 Cleaning work activities have standardized equipment and techniques, however, sometimes they are not adapted to the psychophysiological needs of workers ^(^ 6 ^-^ 7 ^)^ . As a result, the SHL work process has been associated with multiple ergonomic risks capable of predisposing to MSP, with a high prevalence of 70.1% and 25.5% in strong to unbearable intensity ^(^ 7 ^)^ . This scenario is characterized by manual and repetitive activities, little mechanical assistance, muscular effort excess, use of force and intense rhythm ^(^ 6 ^)^ . In addition, cleaning activities are permeated by non-ergonomic body postures that are associated with load handling tasks and frequent static muscle work in pulling, pushing, standing and walking ^(^ 8 ^-^ 9 ^)^ . 

 These recurrent exposures, although avoidable, predispose to musculoskeletal overload, resulting in MSP symptoms ^(^ 7 ^-^ 8 ^)^ . The consequences of severe levels of MSP can be physical, with limitations in carrying out daily activities and in quality of life and emotional, with interference on behavior, mood and sleep ^(^ 10 ^)^ . 

 Given the above, the main perspective of this study is to offer subsidies to health and support service managers on the ergonomic risk factors that trigger musculoskeletal symptoms in hospital cleaning workers. The relevance of studies aimed at the health of SHL workers in institutional daily lives, often demarcated by technical and institutional prejudices and by the relaxation of protection laws, is reiterated ^(^ 11 ^)^ . Studies confirm the invisibility and lack of actions aimed at both health promotion and disease prevention in this population ^(^ 5 ^,^ 8 ^,^ 11 ^)^ . 

 The interface between cleaning workers and nursing occurs due to the increasing role of nurses in managing SHL. Thus, the aim is to maintain a clean, pleasant and safe environment, and to minimize risks, especially those related to infections, which can interfere with care planning and the occupational safety of support and health team professionals ^(^ 11 ^)^ . Therefore, nurses’ commitment to caring for people’s health in all aspects of their lives is reaffirmed, including at work and, above all, in creating healthy work environments. 

To this end, the hypothesis is: SHL workers are exposed to ergonomic risks, especially related to MSP. In this sense, Convergent Care Research makes it possible to collectively build innovations and teaching-learning actions to minimize them. Therefore, the objective was to analyze exposure to ergonomic risks and the occurrence of MSP in SHL workers.

## Method

### Study design

 This study used Convergent Care Research (CCR) ^(^ 12 ^)^ as a methodological approach. This approach is intended for theorizing construction of problems emerging from practice, in order to obtain innovations in care practice ^(^ 12 ^)^ . Furthermore, it allows the integration of various methods, strategies and techniques, from the care practice itself, transforming them into research results ^(^ 12 ^)^ . 

 Therefore, the data production stage was designed based on mixed methods research, using the methodological strategy of the convergent parallel project ^(^ 13 ^)^ . A cross-sectional and an exploratory-descriptive researches were developed. At the end of the study, the results were merged in search of convergences and/or divergences, considering the attribution of equal weight to the two approaches (QUAN + QUAL) ^(^ 13 ^)^ . 

 Regarding compliance with methodological rigor criteria for research writing, the Strengthening the Reporting of Observational Studies in Epidemiology (STROBE) ^(^ 14 ^)^ instrument was used for the quantitative study, and for the qualitative study, the Consolidated Criteria for Reporting Qualitative Research (COREQ) ^(^ 15 ^)^ . In addition to the Equator Network guides, the criteria for mixed studies were followed according to the Mixed Methods Appraisal Tool (MMAT) ^(^ 16 ^)^ . 

### Scenario

Teaching hospital in the central region of the State of Rio Grande do Sul, Brazil.

### Period

Data collection was implemented in two stages: diagnosis (QUAN+QUAL) and “qualitative dive” (QUAL), between July 2019 and September 2020.

### Population

SHL workers working during the data collection period and who met the selection criteria. They were hired by a company providing outsourced services to the teaching hospital to clean and disinfect 403 hospital beds and 30 thousand square meters (m²) of physical area.

### Selection criteria

In the QUAL diagnostic stage, consisting of systematic observation and ergonomic assessment (photographic records), all SHL workers from different sectors and functions participated.

In the QUAN stage, SHL workers working during the period stipulated for data collection were included, with a minimum period of 30 days in the role. The delimitation of this period was established together with the SHL management, taking into account the time needed for admission training, adaptation to the company and to the work sector. Also, so that, after this initial period and during daily cleaning activities, it would be possible to observe the presence of MSP and exposure to ergonomic risks and, therefore, contribute more effectively to the research. Workers absent during the data collection period due to vacation or extended leave (for any reason) were excluded.

In the QUAL stage, of the “qualitative dive”, six convergence groups (CG) were carried out. To this end, availability to join the CG and participation in the prior investigative stage (QUAN) were considered as inclusion criteria.

### Participants

In the first QUAL diagnostic stage, all SHL workers (N=152) participated in the observation and photographic records, regardless of their sector of activity. In the QUAN stage, 149 (98%) SHL workers agreed to respond to the questionnaire. Losses (n=3; 2%) resulted from non-acceptance to participate in the study (n=2) and absence during the data collection period (n=1). In the QUAL in-depth stage, an average of 12 SHL workers and eight managers participated in each CG. Of these, two worked in the management of the hospital’s Hygiene and Waste Management Sector and six in direct supervision of SHL, under a contract with a company providing outsourced services.

### Study variables

Exposure: ergonomic risks. Primary outcome: musculoskeletal pain. Independent variables: sociodemographic, work and health characteristics.

### Instruments used to collect information

In the QUAN stage, the questionnaire was composed of five blocks: block (A) with two questions related to identifying the date of collection and the location of the study. The second block, (B), included five sociodemographic characterization questions: sex; age group; education; marital status and number of children. Block (C) consisted of seven questions about the work profile: shift; working time in the role; daily workload; time for leisure; number of people on the scale; having another job and carrying out training on ergonomic risks. The fourth block, (D), had 11 items evaluating workers’ health: smoking; alcohol consumption; use and indication of medication; medical diagnosis of a disease; daily sleep hours; practicing physical activity and anthropometric measurements (weight, height, waist and hip circumference).

 Furthermore, block (E) comprised the question related to the outcome – MSP (dependent variable). The Nordic Musculoskeletal Questionnaire (NMQ) ^(^ 17 ^)^ in the Brazilian version was used, which makes it possible to verify reports of MSP in ten anatomical regions. In this study, a worker who answered affirmatively to the question: “In the last seven days, have you had any pain or discomfort in (neck, shoulders, elbows, wrist or hand, thoracic spine, lumbar spine, thighs, legs, knees and ankles)?”. In addition, the Corlett and Manenica Diagram was used as an auxiliary tool for diagnosis and for demarcating the intensity of pain or discomfort in each body segment, visualized in the image of the human body ^(^ 18 ^)^ . 

Therefore, the data obtained through systematic observation, photographic records (QUAL) and questionnaire (QUAN) served to support group teaching-learning actions aimed at the demands of those researched (QUAL). To this end, in the last stage, six CG were carried out with a view to deepening the QUAL data regarding the research question: “What is the perception of ergonomic risk of SHL workers and how do they experience exposure to MSP?”.

### Data collection

 The first stage (diagnosis) occurred through systematic observation, photographic records and the questionnaire (sociodemographic, labor, health data, NMQ ^(^ 17 ^)^ and Corlett and Manenica Diagram) ^(^ 18 ^)^ . The second stage (qualitative deepening) occurred through six CG ^(^ 12 ^)^ . 

Thus, in the first QUAL stage systematic observation and photographic records were aimed at mapping ergonomic risks in the work activities of the workers investigated, such as body posture, movements when using equipment, weight lifting and the arrangement of furniture. It was carried out on three shifts, at different times of the working day, in 53 observation shifts, lasting four hours each, totaling 212 hours.

QUAN data collection (questionnaire) occurred during the work shifts of those investigated after due authorization from SHL management. It was carried out by a researcher and previously trained postgraduate students through a “pilot test” of the questionnaire application. Afterwards, the workers were invited individually in the sectors in which they worked. In cases where a worker was absent at the time of data collection, contact was made by telephone to schedule a new date.

 When collecting QUAL data, especially the CG, the days, shifts, strategies and physical space were defined with the participants, in order to guarantee participation and not affect work activities. The CG were guided by a semi-structured script. The discussions and statements were audio recorded on a device and lasted between 1 hour and 1 hour 30 minutes. These recordings were stored on compact disc (CD) and transcribed in full, using Microsoft Office Word ^®^ and inserted into the software NVIVO ^®^ 10, in which data coding and organization were carried out. 

 It is noteworthy that at the end of the sixth CG theoretical saturation was reached ^(^ 19 ^)^ , as there was a repetitiveness of aspects related to exposure to ergonomic risks and musculoskeletal disorders among SHL workers, culminating in the collective construction of the technical-technological product “ *Programa de educação continuada: um olhar sobre a saúde e a ergonomia no trabalho de limpeza* ” (“Continuing education program: a look at health and ergonomics in cleaning work”). 

### Data processing and analysis

 QUAN data were entered into the Epi-info ^®^ program, version 6.04, with independent double entry. Afterwards, errors and inconsistencies were checked. Data analysis was carried out in SPSS ^®^ (Statistical Package for the Social Sciences, SPSS Inc, Chicago), version 18.0, using bivariate statistics. Categorical variables were presented with absolute (N) and relative frequencies (%). Quantitative variables were described by measures of central tendency (mean or median) and dispersion (standard deviation or interquartile range), taking into account the normality or not of the data (Kolmogorov-Smirnov test). 

MSP was analyzed dichotomously (present or absent) in each anatomical region, presenting the absolute (N) and relative (%) frequencies. Bivariate analyzes were carried out to identify associations between MSP and the independent variables. Pearson’s Chi-Squared or Fisher’s Exact test was used considering a statistical significance level of 5% (p<0.05).

**Figure 1:**
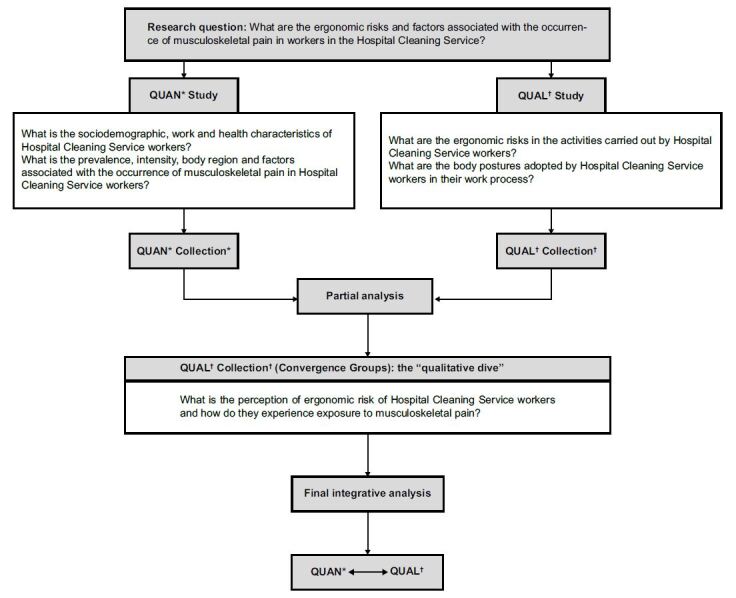
- Diagram representing the methodological trajectory of the study. Santa Maria, RS, Brazil, 2019-2020

 QUAL data was subjected to content analysis ^(^ 20 ^)^ . Especially in the pre-analysis phase of the material, data on the majority and recurring themes in the CG’s observations and speeches emerged. These were arranged side by side with the QUAN data, seeking their fusion and complementarity. Thus, in the partial and final integrative analysis, the information collected was mixed to determine convergences, differences and combinations ^(^ 13 ^)^ ( Figure 1 ). 

*QUAN = Quantitative; ^†^ QUAL = Qualitative

 As the QUAN and QUAL data were collected, they were analyzed. In total, there were 97 pages of raw data, which were treated according to the CCR sequential process (apprehension, synthesis, theorization and transfer) ^(^ 12 ^)^ . 

 The apprehension phase ^(^ 12 ^)^ enabled the process of organization and assimilation of data, which allowed systematization for subsequent integration (QUAN+QUAL) ^(^ 13 ^)^ . The information obtained in the QUAL stage was coded in the field diary and in the CG transcriptions as required by the CCR: NO (observation notes), NQ (questionnaire notes), NG (group discussion notes), NA (assistance notes), ND (diary notes), NM (methodological notes) and NT (theoretical notes) ^(^ 12 ^)^ . 

 The synthesis phase ^(^ 12 ^)^ was implemented using the field diary, photographic records of body postures, results on the prevalence of MSP and associated factors (p<0.05) and statements obtained in the CG. These data were marked chromatically and reorganized according to similarities, consistency of meaning and conceptual relationship ^(^ 20 ^)^ . 

 Furthermore, photographic records of body postures that elucidated exposure to MSP in the body segments prevalent in the quantitative stage were chosen. In these records, what was right or wrong was demarcated, from an ergonomic point of view, based on the body posture adopted when using equipment and work materials ( Figure 4 ). 

 Finally, theorization ^(^ 12 ^)^ occurred by mixing the sources of evidence (QUAN + QUAL) and also by surveying points of convergence and/or divergence based on the integrative analysis of the different approaches. 

### Ethical aspects

Research authorized by the institution and approved by its Research Ethics Committee, under registration no. 2,821,335, in August 2018. The ethical precepts of research involving human beings were respected, in accordance with Resolution 466/12. The anonymity of the participants was preserved, naming them with the acronyms PP (Research Participant) and the sequential numbering. Participants signed the Free and Informed Consent Form and the Authorization Term for image use, in two copies, and were informed about the objectives of the study and the possibility of withdrawing from participation.

## Results

**Figure 2:**
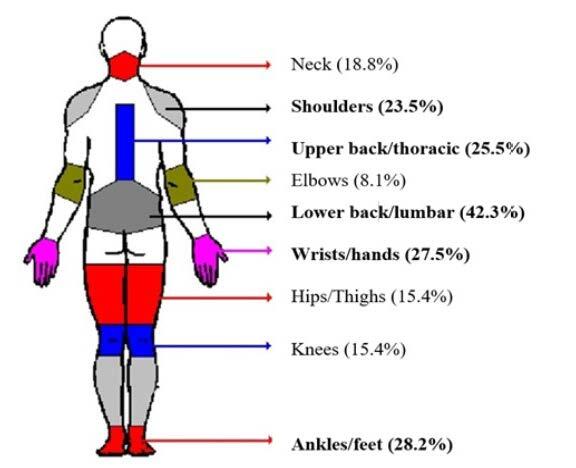
- Musculoskeletal pain according to anatomical location, reported by Hospital Cleaning Service workers in the last 7 days (N=149). Santa Maria, RS, Brazil, 2019-2020

 For this study, due to the average time of 11 months working in the role (59.7%) and to minimize memory bias due to self-report, MSP was considered as that reported in the seven days prior to the research, in the regions described in Figure 2 ^(^ 17 ^-^ 18 ^)^ : 

 In Figure 3 , the joint display is presented with the integration of sociodemographic, work and health factors associated with the occurrence of MSP (p<0.05) in the five prevalent body segments and the ergonomic risk factors highlighted (QUAN + QUAL), through the convergence of research approaches ^(^ 13 ^)^ . 

**Figure 4:**
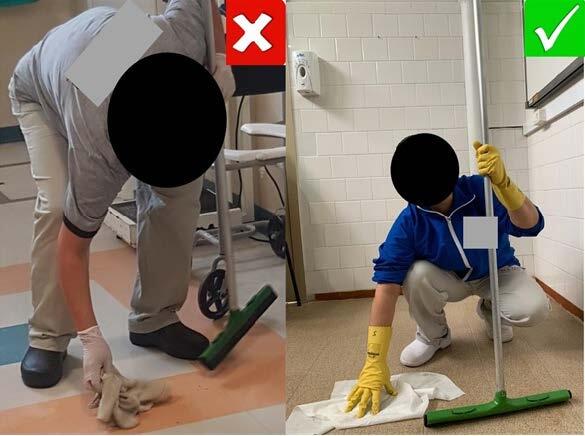
- Ergonomic risks present in the rotation and flexion posture of the trunk and use of equipment not adapted to the psychophysiological needs of workers in the Hospital Cleaning Service. Santa Maria, RS, Brazil, 2019-2020

Source: Research data. Records authorized by workers participating in the study

 The ergonomic assessment identified that the most uncomfortable posture, self-reported by SHL workers, was the standing position with rotation and flexion of the trunk to twist the cleaning cloth, as well as the use of material (squeegee extender) not adapted to their psychophysiological needs (height and demand) ( Figure 4 ). This unfavorable posture requires effort in anterior flexion of the spine and inclination of the trunk (squatting). It is used in most of the participants’ work activities, such as handling the bucket, removing the garbage bag and wringing out the cloth. There is a significant ergonomic risk related to the symptoms of MSP in the lumbar and thoracic spine, as shown in Figure 4 . 

 At the end of the integrative analysis, it was evident that SHL workers are exposed to multifactorial ergonomic risk factors related to the occurrence of MSP ( Figure 3 ). Among them, uncomfortable, limited, asymmetrical, repeated and/or prolonged working postures stand out, as well as repetitive movements, prolonged orthostatism and the use of equipment not adapted to the psychophysiological needs of workers. This can overload the tissues and exceed their stress limits, causing tissue damage due to inadequate efforts and overload on the body’s musculoskeletal structures, especially the spine ( Figure 4 ). 

**Figure 3:** - Joint display of the integrative analysis with the mix of ergonomic risk factors associated with the occurrence of musculoskeletal pain among Hospital Cleaning Service workers (n=152). Santa Maria, RS, Brazil, 2019-2020

Prevalent body region of MSP ^*^ , in the last 7 days:	**QUAN** ^†^ **Results**	**QUAL** ^‡^ **Results**			**Convergences**
	**Factors associated with the occurrence of MSP** ^*^ **:**	**Observation**	**Photographic records**	**Convergence Groups**	
**Lower back (42.3%)**	Self-medication (p=0.020) ^§^ .	– Practice of self-medication for MSP ^*^ carried out before the start of the work shift; – Use of the squeegee with the need to squat, dip the cloth in the bucket, stand up, bend over and wring the cloth.	– Standing posture with trunk rotating; – Posture of anterior flexion of the spine and inclination of the trunk to wring out the cloth, pick up the bucket or remove the garbage bag; – Squeegee extender not adapted to the worker’s psychophysiological needs.	– Symptoms of lower back pain during work activities with the use of floor cleaning equipment (washing machine and mop); – Use of analgesic and anti-inflammatory medications for MSP symptoms ^*^ .	Self-medication is used routinely and preventively to alleviate the symptoms of MSP ^*^ , without recognizing the risks inherent to this practice.
**Ankles or feet (28.2%)**	– Sedentarism (p=0.041) ^§^ ; – Sleep less than eight hours a day (p=0.039) ^§^ .	– Prolonged orthostatism; – Inadequate workstation furniture and lack of swivel casters; – High physical demand; – Improperly distributed shifts and workload; – Lack of scheduled breaks between work tasks; – Repetitive efforts.	– Use of inappropriate protective footwear (straight base, without elevation and with heat and moisture retaining insoles); – Repetitiveness of movements; – Workplace furniture not adapted to the psychophysiological needs (height and demand) of cleaning workers working in the sector, with the absence of swivel casters to reduce effort.	– Difficulty taking breaks at work and raising the lower limbs; – Lack of appropriate environment for the planned interval; – Dynamic activity; – Overweight/obesity; – Workplace furniture not adapted to the needs of cleaning workers in the sector. – Use of non-functional/uncomfortable protective footwear.	– Practicing physical activity can be a “protective factor” for workers not to report MSP ^*^ ; – Workers who sleep less than eight hours a day have a higher prevalence of MSP ^*^ in this region. Qualitative data confirm this association, due to the insufficient rest reported.
**Wrists or hands (27.5%)**	–Ex-smoking (p=0.015) ^§^ ; – Use of some type of medication (p=0.004) ^§^ .	– Inadequate use of equipment (squeegee extenders, mop and washing machine) not adapted to the psychophysiological needs (height and demand) of workers; – Force used in twisting the cloth; – Difficulty adapting to replacing the cleaning technique with the use of cloths and buckets with the use of a multipurpose mop.	– Wrist flexion posture when using cleaning equipment and materials; – Constant physical pressure from hands on work objects; – Constant use of upper limbs to clean and disinfect surfaces, benches and equipment.	– Use of equipment with fixed extenders/cables that are not adjustable to the worker’s psychophysiological needs (height and demand); – Use of analgesic and anti-inflammatory medications to live with MSP ^*^ in wrists and hands.	– Former smokers have a higher prevalence of MSP ^*^ in the wrists or hands. This significant association was reaffirmed in the observations and statements of the convergence groups; – Workers use analgesic and anti-inflammatory medications for MSP ^*^ on their wrists or hands. This finding converges with the high prevalence of pain in these regions in the last seven days (38.6%).
**Upper back/thoracic spine (25.5%)**	There was no statistically associated factor (p<0.005) ^§^ .	– Rapid repetitive movements when cleaning under hospital beds; – Requirement of flexion of the vertebral trunk when cleaning horizontal surfaces.	– Flexion and rotation posture of the spine required in cleaning activities; – Manual lifting and transport of weight (garbage bag).	– Neutral postures and repetitive movements of the upper limb joints; – Trunk flexion posture when using the multipurpose mop.	There was no statistically associated factor (p<0.005) ^§^ to analyze convergence.
**Shoulders (23.5%)**	Self-medication (p=0.026) ^§^ .	– Posture with arms raised, without support; – Elbow extension posture in shoulder abduction or elevation.	– Non-ergonomic body posture when handling the rotary washing machine; – Arm position above shoulder height for cleaning ceilings and vertical surfaces.	– Usual practice of self-medication to relieve MSP ^*^ symptoms in the shoulders.	Convergence of QUAN ^†^ +QUAL ^‡^ research approaches on the routine practice of self-medication, with the use of analgesics and anti-inflammatories by the workers investigated.

* Musculoskeletal pain;

† Quantitatives;

‡ Qualitatives;

§ Pearson’s Chi-Squared Test

**Figure 5:**
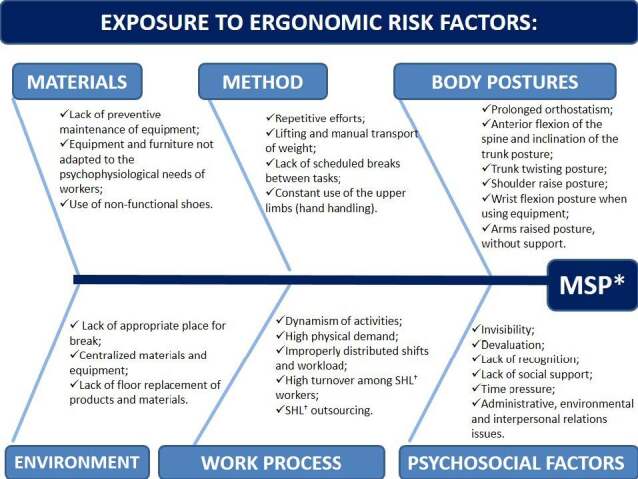
- Diagram of exposure to ergonomic risk factors related to the occurrence of musculoskeletal pain in Hospital Cleaning Service workers. Santa Maria, RS, Brazil, 2019-2020

 In this way, there is the occurrence of MSP, which means that the impact is especially pronounced when there is a combination of two or more of these risk factors in a single activity ( Figure 3 ). Added to this are the conditions existing in the work process, in environmental and in psychosocial factors predisposing to MSP in SHL, as illustrated in Figure 5 below: 

*Musculoskeletal pain; ^†^ Hospital Cleaning Service

 The mixing of data, through the convergent parallel project, made it possible to compensate the weaknesses inherent to one method with the strengths of the other, producing better substantiated and validated results ^(^ 13 ^)^ regarding exposure to ergonomic risks and the occurrence of MSP in SHL workers, as shown in Figures 3 to 5, which constitute the focus of the discussion. Furthermore, the presence of psychosocial factors was found in the analyzed population, favoring exposure to ergonomic risk and the development of MSP ( Figure 5 ). 

## Discussion

 The results obtained regarding the main location of MSP, the lumbar region, converge with the epidemiological data published to date ^(^ 7 ^,^ 21 ^-^ 23 ^)^ . Among them, the National Health Survey ^(^ 21 ^)^ identified that around 34.3 million (21.6%) of Brazilians presented symptoms of chronic back pain and, in addition, 2.5% were diagnosed with “ *Distúrbios Osteomusculares Relacionados ao Trabalho* ” (DORT in Portuguese) *-* Work-Related Musculoskeletal Disorders (WMSD). Furthermore, in more than half of the cases the pain was of high intensity and chronic, that is, lasting more than 6 months ^(^ 7 ^)^ . 

 Systematic review and meta-analysis on the effectiveness of digital self-care for pain and functional disability in people with spinal MSP also identified a predominance of the lumbar region. A high prevalence (90.9%) of chronic pain was found, with a negative impact on functional capacity ^(^ 22 ^)^ . 

 When considering the prevalence and multifactorial origin of lumbar symptoms ^(^ 22 ^)^ , a study identified a relevant correlation with inadequate postures, manual lifting, flexion, twisting and tilting of the spine with repetitive movements ^(^ 23 ^)^ . Besides, repetitive ergonomic tasks can place an inappropriate burden on the musculoskeletal elements of the spinal column, which can result in MSP in the lumbar spine ^(^ 22 ^-^ 23 ^)^ . 

 SHL workers perform occupational activities predominantly standing, 94% of the time ^(^ 23 ^)^ . Therefore, exposure to MSP in the region of the ankles or feet, according to the body segment prevalent in MSP, is related to prolonged orthostatism, which is a convergent ergonomic risk factor in the mix of data in this study. Therefore, maintaining a standing posture for a period of at least two hours requires continuous contraction of the muscles responsible for supporting this position, which can result in overload of the spinal column ^(^ 23 ^)^ . Additionally, it tends workers to use the lower limbs asymmetrically and alternately as support to facilitate blood circulation and reduce compression on the joints, making them more susceptible to fatigue, reduced venous return and MSP in this segment ^(^ 23 ^)^ . 

 In this context, the hospital setting has characteristics of the environment and work organization that increase the likelihood of SHL workers reporting complaints of MSP in different body segments. In the ankles and plantar surface of the feet, the absence of designated places to sit during breaks and scheduled breaks at work, in addition to the need to walk long distances, constitute important risk factors for the symptoms of MSP ^(^ 6 ^,^ 24 ^)^ . The use of safety footwear at work, with conventional polyurethane and ethylene-vinyl acetate materials, is also associated with the occurrence of symptoms in this segment ^(^ 25 ^)^ . These materials reduce breathability, causing heat and moisture retention and it is recommended that attention be paid to the microclimate of the footwear, with the use of permeable textile insoles ^(^ 25 ^)^ . 

 The practice of physical activity was configured as a “protective factor” for SHL workers not to report MSP in ankles or feet, since sedentary workers had a high prevalence of musculoskeletal symptoms in this segment. This can be explained because workers who do not practice physical activity regularly tend to not be physically prepared for highly difficult demands. Furthermore, a sedentary lifestyle and immobility are factors that increase the stiffness of tendons, fascia, ligaments and muscles. This condition causes, among other disorders, muscular and supporting skeletal tissue atrophy, increased myofascial rigidity, somatosensory deficits and, linked to these factors, MSP ^(^ 26 ^)^ . 

 The results of this study corroborate the literature regarding the consequences of a sedentary lifestyle. In this case, body weight tends to increase and cause excessive pressure on the plantar surface of the feet, conditioning the perception of pain and discomfort in the lower limbs for the continuous support of body weight ^(^ 26 ^)^ . The aggravating factor is the possibility of a “vicious circle”, in which the individual with excess body weight and symptoms of MSP gradually reduces the practice of physical activities, subsequently perpetuating their obesity ^(^ 26 ^)^ . Besides, obesity is one of the main risk factors for osteoarthritis, overload of the thoracolumbar spine and MSP in the lower limbs ^(^ 26 ^-^ 27 ^)^ . 

 Former smokers had a higher frequency of MSP in the wrist or hand region in this study. A similar perception is found in the Brazilian literature, pointing out that the probable signs of this association may be related to the change in pH and the nutrition of the intervertebral discs by the components of cigarettes, predisposing to herniations. Furthermore, nicotine affects the central nervous system and causes hypoxia, vasoconstriction, changes in fibrinolysis with a decrease in cellular oxygenation and other mechanisms that harm the nutrition or structure of muscle tissue, interfering with the perception of pain ^(^ 28 ^)^ . 

 Among the factors that can influence the occurrence of injuries during the use of hands are the weight and type of load ^(^ 29 ^)^ . When the shape of the load or equipment approaches the anatomy of the hands, greater contact with the object is provided, allowing for greater grip. In this case, a smaller amount of force is used, making the process easier. Likewise, a larger object requires more force to maintain it and a greater number of body segments to stabilize it ^(^ 29 ^)^ . This last situation occurs when handling the mop and washing machine, recorded in the ergonomic assessment of this research. This happens because the mop handle is fixed, that is, it does not have an adjustable extender and, furthermore, the grip location requires strength when handling. 

 The flexion posture and the rotation of the spine occurred during cleaning work, associated with lifting weights (garbage bags), which constituted an aggravating factor in relation to the occurrence of MSP in the thoracic spine. This uncomfortable, limited and asymmetrical posture is used to clean under hospital beds, with rapid repetitive movements ^(^ 29 ^)^ . 

 The fact that SHL workers remain, for extended periods, with their arms raised, without support, was evidenced as an important risk factor for MSP in the shoulder region. This is because keeping the arms above the height of this segment, such as when cleaning surfaces, walls and ceilings vertically, causes fatigue in the shoulder and biceps muscles, creating a greater risk of injury and musculoskeletal impairments, especially tendonitis ^(^ 30 ^)^ . Moreover, MSP in the shoulders can be justified by substantial physical effort with repetition of movements, as well as by the gestural biomechanics characteristic of the activity of sweeping, in addition to handling cargo and placing trash in the functional cart ^(^ 30 ^-^ 31 ^)^ . 

 A study with the janitorial staff of a public university in Malaysia, composed of supervisors, landscapers and cleaning workers, identified the shoulder region as having a prevalence of moderate MSP (71.6%). The severity of symptoms was associated with working time of more than three years (p<0.001) and with the implementation of measures to control and prevent musculoskeletal symptoms (p=0.018) ^(^ 31 ^)^ . These measures included motorized cleaning machines and equipment, mechanical assistance for moving heavy loads, training on work ergonomics, rotation of workers in daily work activities, sufficient rest time, and regular dialogues with supervisors for imminent issues ^(^ 31 ^)^ . 

 Furthermore, in the perception of SHL workers, ergonomic risk and MSP were influenced by the psychosocial aspects of work. In this case, ergonomic risks are not limited to frequent mechanical exposures, unfavorable body postures and the use of equipment, but organizational, psychological and social issues can also constitute risk factors for MSP ^(^ 6 ^,^ 32 ^)^ . 

 Therefore, risks, aspects or psychosocial factors exist from the interaction between the workplace, organizational and environmental conditions, skills and individual needs of the worker ^(^ 32 ^-^ 33 ^)^ . These contacts can lead to tension (high demand and low level of control), stress, lack of recognition, social devaluation of work, loss of motivation and engagement, gender inequality and lack of social support, depending on the way cleaning workers experience them ^(^ 6 ^,^ 33 ^)^ . The type of management and leadership may or may not enhance these negative effects on performance, job satisfaction and health, especially regarding the risk of MSP ^(^ 32 ^-^ 33 ^)^ . 

 However, it is recognized that pain is an unpleasant sensorial and emotional experience, which is conditioned by the subjectivity of those who suffer it ^(^ 34 ^)^ . Multiple factors were involved in the occurrence of MSP in SHL workers in this study, from symptoms in segments of the locomotor system that trigger nociceptive pain to others of a psychosocial nature, such as high work demand, limited control and little support from supervisors and colleagues ^(^ 33 ^-^ 34 ^)^ . 

 In fact, recent investigations support the multifactorial nature of exposure to ergonomic risks, as well as the influence of psychosocial factors in triggering MSP, which is why it requires a biopsychosocial and interdisciplinary approach ^(^ 6 ^,^ 33 ^-^ 34 ^)^ . To this end, prevention and management strategies, individual and collective, targeted and appropriate for minimizing exposure to ergonomic risks need to take into account biological, psychological and sociodemographic determinants, health and life habits, as well as results regarding the effects from MSP ^(^ 6 ^,^ 22 ^)^ . 

 In the meantime, psychosocial risk factors are related to a worse perception of health, and early detection becomes crucial for the management of MSP. They are considered “yellow flags”, that is, predictors of non-return to work, transition from acute to chronic pain and work incapacity, on which strategies aimed at SHL workers must focus ^(^ 32 ^)^ . 

 The use of quantitative and qualitative methods made it possible to minimize the inherent weaknesses of both, as the positive points of one approach compensated for those of the other ^(^ 13 ^)^ . The limitations concern the small sample size and little heterogeneity both in the characteristics of SHL workers and in the variability in relation to the cleaning activities carried out. Research with longitudinal and experimental designs, including larger samples and other health care scenarios may generate other information about the phenomenon investigated. 

 As a contribution to science and the field of worker health, the aim was to analyze the modifiable ergonomic risk factors in cleaning activities and the body segments affected by MSP in support workers, such as SHL and, above all, to facilitate the process of transformation and the promotion of healthy work environments. Scientific evidence can contribute to the reorganization of the SHL environment, process and work organization, with the aim of minimizing the physical overloads to which they are exposed ^(^ 6 ^)^ . Additionally, it is necessary that the management of the institutions in which SHL operates also implement actions with the aim of promoting the due recognition and visibility of these workers, given the influence of psychosocial aspects in the occurrence of musculoskeletal symptoms, evidenced in this study. 

 It is considered that the innovation of this study lies in the use of Convergent Care Research as a “guiding thread” of research in the field of cleaning worker health, ranging from the initial stage, known as “conception”, to the final “transfer”. Within this movement, mixed methods research was incorporated considering that a methodological approach alone would not explain the complex, multicausal and subjective exposure to ergonomic risks and MSP. In this process, especially in the CG stage, a strategy was collectively constructed with recommendations for improvements to the process and organization of cleaning work, with a view to minimizing exposure to ergonomic risks and the outcome of MSP. This was titled “ *Programa de educação continuada: um olhar sobre a saúde e a ergonomia no trabalho de limpeza* ” (“Continuing education program: a look at health and ergonomics in cleaning work”), developed through teaching-learning actions. 

## Conclusion

Self-perception of exposure to ergonomic risks and MSP was consistent with the multifactorial nature obtained in the study’s sources of evidence. These indicate that SHL workers are exposed to modifiable ergonomic work risks, which in turn are associated with the occurrence of MSP symptoms, predominantly in the lower back, ankles or feet, wrists or hands, upper back and shoulders. Furthermore, in the population studied, exposure to ergonomic risk and the triggering of MSP are influenced by the psychosocial aspects of work. Given this panorama, the hypothesis of this study was confirmed.
